# Advancements in Medical Radiology Through Multimodal Machine Learning: A Comprehensive Overview

**DOI:** 10.3390/bioengineering12050477

**Published:** 2025-04-30

**Authors:** Imran Ul Haq, Mustafa Mhamed, Mohammed Al-Harbi, Hamid Osman, Zuhal Y. Hamd, Zhe Liu

**Affiliations:** 1 School of Computer Science and Communication Engineering, Jiangsu University, Zhenjiang 212013, China; 1000006380@ujs.edu.cn; 2 College of Information and Electrical Engineering, China Agriculture University, Beijing 100083, China; mustafamhamed@cau.edu.cn; 3 Medical Imaging Department, King Abdullah bin Abdulaziz University Hospital, Riyadh 11552, Saudi Arabia; moalharbi@kaauh.edu.sa; 4 Radiological Sciences Department, College of Applied Medical Sciences, Taif University, Taif 21944, Saudi Arabia; ha.osman@tu.edu.sa; 5 Department of Radiological Sciences, College of Health and Rehabilitation Sciences, Princess Nourah bint Abdulrahman University, P.O. Box 84428, Riyadh 11671, Saudi Arabia; zyhamd@pnu.edu.sa

**Keywords:** multimodal machine learning, radiology, medical image and text, fusion, translation, representation learning

## Abstract

The majority of data collected and obtained from various sources over a patient’s lifetime can be assumed to comprise pertinent information for delivering the best possible treatment. Medical data, such as radiographic and histopathology images, electrocardiograms, and medical records, all guide a physician’s diagnostic approach. Nevertheless, most machine learning techniques in the healthcare field emphasize data analysis from a single modality, which is insufficiently reliable. This is especially evident in radiology, which has long been an essential topic of machine learning in healthcare because of its high data density, availability, and interpretation capability. In the future, computer-assisted diagnostic systems must be intelligent to process a variety of data simultaneously, similar to how doctors examine various resources while diagnosing patients. By extracting novel characteristics from diverse medical data sources, advanced identification techniques known as multimodal learning may be applied, enabling algorithms to analyze data from various sources and eliminating the need to train each modality. This approach enhances the flexibility of algorithms by incorporating diverse data. A growing quantity of current research has focused on the exploration of extracting data from multiple sources and constructing precise multimodal machine/deep learning models for medical examinations. A comprehensive analysis and synthesis of recent publications focusing on multimodal machine learning in detecting diseases is provided. Potential future research directions are also identified. This review presents an overview of multimodal machine learning (MMML) in radiology, a field at the cutting edge of integrating artificial intelligence into medical imaging. As radiological practices continue to evolve, the combination of various imaging and non-imaging data modalities is gaining increasing significance. This paper analyzes current methodologies, applications, and trends in MMML while outlining challenges and predicting upcoming research directions. Beginning with an overview of the different data modalities involved in radiology, namely, imaging, text, and structured medical data, this review explains the processes of modality fusion, representation learning, and modality translation, showing how they boost diagnosis efficacy and improve patient care. Additionally, this review discusses key datasets that have been instrumental in advancing MMML research. This review may help clinicians and researchers comprehend the spatial distribution of the field, outline the current level of advancement, and identify areas of research that need to be explored regarding MMML in radiology.

## 1. Introduction

The accelerated development of science and technology is revolutionizing people’s lives worldwide. Numerous novel innovations have significantly influenced scientific investigations in the 21st century [1]. Out of all these breakthroughs, machine learning (ML) methods are the most renowned and extensively used. ML is not a recent technology, since it combines computation and statistics [2]. The increasing availability of information from numerous sectors has created opportunities for the extensive use of ML in education, banking, economics, smart cities, and healthcare.

Moreover, swarm intelligence techniques are extensively utilized and implemented to address various optimization challenges in machine learning (ML) [3]. ML is a comprehensive scientific technique that includes several classes of approaches. It may be taught and improved to generate precise predictions using the knowledge and data available in its environment, which may be retrained and used when application contexts and data sources change.

**Figure 1:**
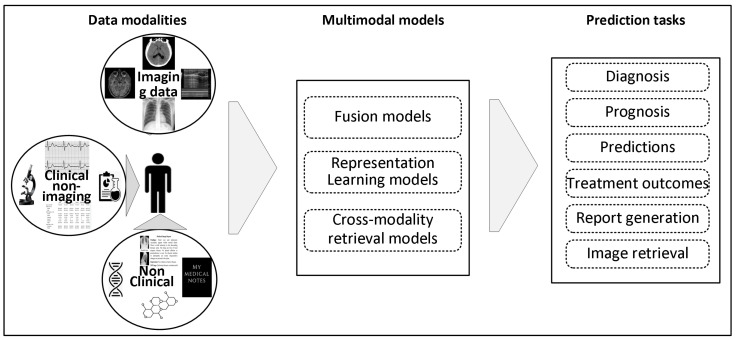
The conceptual pipeline of MMML in radiology. This figure illustrates how diverse clinical and non-clinical data modalities are processed through specific fusion and learning strategies to support a variety of clinical prediction tasks. It provides a narrative backbone for this review’s structure and future directions.

In clinical contexts, several kinds of data from multiple sources provide patient information. This encompasses radiographic images, such as ultrasound, X-ray, CT, MRI, molecular imaging, and nuclear medication. A substantial number of non-imaging details linked to each patient are also gathered, including reports from radiologists, lab examinations, ECG and EEG, etc. In addition, a patient may be linked to non-clinical data such as demographic details, genetic details, and therapeutic histories. Diagnosing an individual commonly requires the healthcare professional to gather and analyze information from various resources (Figure 1). In the medical context, “multimodal data” refers to data beyond simply the imaging modality.

Radiology images comprise a substantial proportion of medical data and have a crucial impact on diagnosing patients. Radiologists are often responsible for summarizing the information found in these images to help doctors make clinical decisions. Understandably, the number of radiological tests has steadily grown with time. Hence, the aforementioned rise and the recognized scarcity of radiologists [4] underscores the need for computer-assisted supporting technologies to analyze radiology images automatically. As a result, medical image processing has become a dynamic sub-field of computer science. Current progress in ML has further advanced research in developing supporting technologies that rely on medical images [2]. Furthermore, these technologies are gradually integrated into healthcare applications [3].

The discipline of ML, which focuses on analyzing data from multiple sources, is called multimodal machine learning (MMML). It has shown significant growth as a sub-domain within computer vision and ML. MMML is similar to how humans process physiological inputs, like vision and sound. It is utilized in various domains, including autonomous driving (using a series of images, radar, lidar, and other sensors), conditioned image creation (using an image and text), audio–visual speech identification (using a series of audio clips and images), visual question answering (using an image and text), and more. The final objective in these situations is to create a feature representation that combines the data from all input sources.

**Figure 2:**
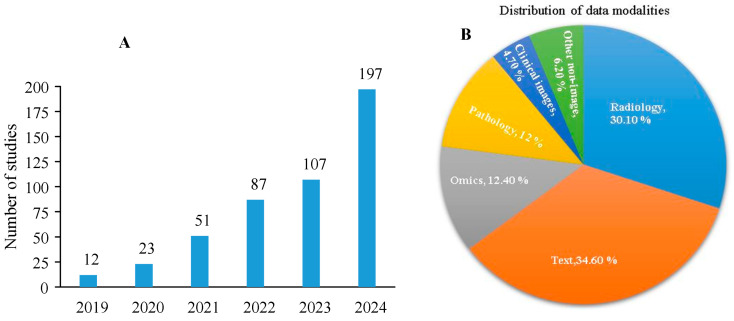
Summary of data modalities utilized in the reviewed articles. (**A**) Bar chart illustrating the exponential growth in the number of studies published annually from 2019 to 2024. (**B**) Pie chart depicting the distribution of various modality groups and the corresponding data modalities employed in the studies.

Recent findings indicate a significant surge in research concerning multimodal AI, especially within healthcare and radiology, from 2019 to 2024 (Figure 2A). This rapid increase highlights the growing interest in using multimodal strategies to improve healthcare decisions. An analysis of data modalities in these investigations reveals that radiography and text are the predominant pairings, followed by omics and pathology (Figure 2B). This trend signifies the growing acknowledgment of MMML as a revolutionary methodology in medical research. This research examines improvements in MMML applied to medical radiology, motivated by the increasing trend and the distinctive potential of merging radiology images with complementary non-imaging data.

The usage of ML has been examined in numerous research efforts, specifically focusing on the advancement of deep learning methodologies across diverse disciplines. These studies have covered a vast collection of subjects, such as overall applicability [5,6,7], image-based methods [8], and a concentrated emphasis on the chest area [9,10]. Within the realm of multimodal usage, several review articles have addressed the application of agnostic methodologies [11], whilst others have highlighted the significance of fusion in identifying diseases [12]. Many reviews have examined models and architectures, focusing on their optimization tactics generally [13], and particularly in the healthcare sector [14,15,16,17,18,19]. Some papers have examined the prospective utilization of MMML, frequently emphasizing the incorporation of image modality within the multimodal framework [20,21,22,23]. Additional evaluations have focused on self-supervised learning, reflecting advancements in that domain [24,25,26,27].

In medical settings, a computer-based assistance mechanism must be capable of processing multimodal data, including radiology images, to make medically practical decisions. This paper focuses on using MMML in radiography and is particularly interested in studies that use medical imaging in conjunction with other types of complementary non-imaging data. The term “multiple modalities” includes both imaging and non-imaging data. Data from various imaging modalities, such as X-ray, ultrasound, MRI, and CT scans for the same individual, belong to a single modality, namely, the imaging modality, for this study.

This review is intended to provide a comprehensive analysis by doing the following:

❖ Examining how integrating medical imaging (X-ray, MRI, CT) and non-imaging (text, ECG, EHR) data improves diagnostic precision, addressing the shortcomings of single-modality machine learning approaches.
❖ Investigating cutting-edge multimodal machine learning approaches, including modality fusion, representation learning, and cross-modality translation, to enhance medical diagnosis and treatment.
❖ Identifying obstacles in implementing multimodal AI, underscoring prospective research possibilities, and emphasizing the need for sophisticated, multimodal diagnostic systems in healthcare.

This paper delineates integrating and interpreting multimodal data by machine learning models to enhance clinical decision-making, as seen in Figure 1, facilitating diagnosis, prognosis, and treatment planning. To provide the groundwork for this topic, we start exploring the main categories of data modalities used in radiography.

## 2. Data Modalities

Figure 1 illustrates several data modalities used in medical treatment [28]. This encompasses image, textual, time series, and tabular data. Acknowledging the importance of data choices in multimodal ML, a review of the primary input modalities utilized for research practice will be presented in this section.

### 2.1. Medical Image Data

Usually, medical imaging data in medical practice are saved in DICOM format as 2D slices [29]. This encompasses clients’ metadata, the specifics of the imaging technique, the data on the instrument utilized for the image, and the parameters of the imaging procedure. Clinical 3D volume imagery is often created using a series of 2D slices of specified dimensions, depicting a particular area of concern in the body. Each slice may be examined and processed alone (2D) or cumulatively (3D) to determine significant details.

In the preparation of medical imaging data for ML, the DICOM format is either transformed into widely utilized imaging types (JPEG, PNG) or converted into the Neuroimaging Informatics Technology Initiative (NifTi) format [30], which is a specialized medical image processing plan that retains vital metadata alongside the image in the file’s header.

#### 2.1.1. Medical X-Ray

X-ray imaging is a readily accessible and cost-effective two-dimensional imaging modality. In 2022, over 50% of the 43.3 million diagnostic examinations conducted in the UK were X-ray scans, establishing X-ray as the predominant medical diagnosis tool [31]. It is based on the idea of differential retardation in X-rays when they pass through different kinds of bodily tissues [32]. Conventional X-rays define five stages of degradation: metal, bone, soft tissue, fat, and air. In X-rays, air manifests as the darkest region, owing to its low density, enabling most X-rays to navigate it, whereas metal, with much higher density, looks dazzling white as it absorbs most of the X-ray beam’s energy. Different shades of gray are seen in fat, soft tissue, and bone tissue; fat appears darker than soft tissue, while bone tissue appears lighter [32].

#### 2.1.2. Computed Tomography

Computed Tomography (CT) provides intricate cross-sectional scans of different body parts [33]. The scans produce three-dimensional image volumes by assembling several consecutive two-dimensional slices from radiography images from various angles. Hounsfield Units (HU), which represent CT scans, are closely correlated with signal attenuation brought on by tissue density relative to water [34]. CT scans are a prevalent option for healthcare diagnosis, providing high-resolution scans, extensive accessibility, affordability, and rapid execution. Nevertheless, they have limits in differentiating soft tissues and exposing people to ionizing radiation [34].

#### 2.1.3. Magnetic Resonance Imaging (MRI)

In contrast to the earlier-mentioned imaging methods, MRI is a non-ionizing technology [35]. The individual is positioned inside a strong magnetic field, which causes the body protons’ magnetic moments to align with the field. Radiation from short radio frequency bursts causes the protons to re-align with the magnetic field. MRI quantifies magnetization in transversal and longitudinal directions, allowing for tissue-specific reconstructions [35]. MRI preserves a higher SNR and offers a comprehensive envision [36]. Nevertheless, this can result in transverse aliasing distortions, particularly during two-dimensional magnetic resonance sampling techniques. MRI data might require anti-aliasing pre-processing procedures, such as filtration or specialized ML algorithms, to reduce these distortions [36]. MRI effectively delivers detailed images of the body’s interior structures, organs, and soft tissues deprived of ionizing radiation [37].

#### 2.1.4. Nuclear Medicine Imaging

Single-Photon Emission CT (SPECT) and Positron Emission Tomography (PET) are nuclear medicine imaging (NMI) procedures that detect gamma photons from radioactive tracers, providing insights into blood flow/function and metabolic activity [38]. In every scan, these approaches produce several 2D slices, ranging from dozens to hundreds [39]. The slices are uniformly distributed, guaranteeing a constant distance throughout the scan. The intensity in the scans is approximate, and every scan generally comes with a corresponding attenuation correction CT image. The primary constraints with these scanning techniques are prolonged collection durations relative to CT, and are often worse quality in comparison to MRI or CT.

#### 2.1.5. Ultrasonography

Ultrasound imaging employs sound radiation at over 20 kHz to detect anatomical features. The data generally include a sequence of 2D frames, although current developments also allow for 3D and 4D scans [40]. Transducer frequency affects image quality, lower frequencies provide deeper depths at the cost of visibility, and higher frequencies offer higher quality but reduced penetration, making them ideal for surface structures. Tissue echogenicity (ability to reflect sound waves) is represented by image brightness, with various degrees of gray indicating various densities [41]. To employ ML approaches, ultrasound imaging is pre-selected, since the spatial position or orientation is not instantly evident and depends upon the machinist technique [42]. Selecting frames and determining regions of interest (ROI) may be performed manually or automatically. Ultrasound data have intrinsic speckling, which may need adjustment during pre-processing [43].

### 2.2. Non-Imaging Data

Non-imaging data provide crucial context and Supplementary Information that can enhance the interpretation of imaging data. Non-imaging data encompass a variety of data types, including text data (unorganized data) like clinical reports and patient histories, time series data (organized data) like ECG, EEG, oximetry, blood pressure, etc., and discrete data (organized data) such as lab results, genetic information, and demographic details.

#### 2.2.1. Text Data

The text modality is an important and widely used health datum in medical treatment. Numerous patient-specific text modes are used in medical treatment [44,45]. This encompasses procedure notes and detailed clinical records produced by healthcare practitioners, including status updates and consultation records. Prescription notes include comprehensive prescription protocol directives, including medicinal product names, doses, and intake requirements [46]. Clinical discharge records are essential throughout a patient’s care, record a patient’s stay in the hospital, and include crucial details such as diagnosis, medication, and after-discharge instructions [47]. Reference notes act as a means of communication across medical practitioners, consisting of relevant patient information and the reasoning for referrals. Radiology reports include detailed accounts of results derived from diagnostic imaging examinations [28,48,49].

Natural language processing (NLP) methods were recently applied to extract essential details using healthcare records [50,51]. This information may be transformed upon extraction into a structured format suitable for application to ML algorithms. Traditional NLP technologies have a significant barrier due to the need for subject-matter specialists to annotate relevant characteristics in textual resources, which is labor-intensive. Diverse methods have been adopted to overcome this obstacle, including active learning strategies for prioritizing textual data for labeling [52]. Different approaches like data augmentation, transfer learning, and the utilization of synthesized medical reports provide more possibilities in this domain [53].

#### 2.2.2. Structured Data (Time Series and Discrete Data)

Structured data refers to spreadsheet-like data presented as a time series or as tabular values concerning the patient’s state. Time series data represent data points gathered at regular periods. The prevalent representations are electrocardiogram (ECG) and electroencephalography (EEG). Fetal surveillance involves assessing a fetus’s health throughout pregnancy and labor, identifying any discomfort or abnormalities by continuously monitoring heartbeat and other parameters [54]. Monitoring cranial tension in individuals with problems like traumatic brain damage may assist in maintaining brain pressure inside acceptable levels [55]. Additional methods consist of respiration inspection [56], oximetry [57], and continuous blood pressure measurement [58]. The ongoing nature of these approaches provides doctors with a dynamic perspective on physiological indicators, facilitating timely response and assessment of treatment effects. Time series data are frequently used to identify early indicators of client deterioration or recovery, forecast results, and establish suitable interventions and therapeutic strategies. Several studies have employed time series data for diverse illness predictions in their frameworks [59,60,61,62].

Discrete data may be shown in a tabular format with columns and rows, often including a single statistic for each attribute per person. It contrasts with time series statistics that, while frequently presented in a tabular style, provide continuous details throughout the period for each individual. Discrete data provide statical representation, but time series give a dynamic perspective on clinical or physiological features. In medical care, the precise organization and assessment of different patients’ information frequently relies on tabular data. Demographic data, including a patient’s sex, age, and race, are commonly presented in a tabulated style and offer a critical background for personalized therapy [28,63]. Health grading procedures, such as standardized evaluations containing quality-of-life measures (e.g., EQ-5D), pain ratings (e.g., KOOS), and illness intensity ratings (e.g., APACHE II), provide measurable data on an individual’s status and therapeutic effectiveness. Medical laboratory statistics, including blood chemistry assessment, are prevalent examples that may enhance the precision of patient status evaluations and inform prognosis [63]. Pharmaceutical data, including drug doses, compositions, and pharmacologic characteristics, are frequently presented in a tabular style and are essential for treatment monitoring and investigation [64]. In addition to these fundamental components, supplementary layers of tabular data may contain treatment schedules, social and behavioral details, medical records, clinical results, and monitoring information [47].

The variety of data, including image, text, and structured data, is the foundation of multimodal machine learning systems. Comprehending these modalities is essential before examining their algorithmic fusion and interpretation.

The following section presents a classification that facilitates the organization of current MMML approaches according to data and methodological strategies, along with further preliminaries for this study.

## 3. Preliminaries

This section builds on the data modalities discussed earlier by organizing MMML efforts into a taxonomy that reflects their methodological structures and fusion strategies, as aligned with the overview in Figure 1.

### 3.1. Data Modalities-Based Classification

In the realm of MMML in radiology, data modalities can broadly be categorized into imaging and non-imaging. Imaging modality is the cornerstone of radiological analysis and includes the traditional forms discussed above (Section 2). Non-imaging consists of various data types, i.e., text data like clinical reports and patient histories, as well as structured data such as time series and discrete data. This leads to two sets of modalities: imaging with text data and imaging with structure data.

### 3.2. Methodology-Based Classification

**Figure 3:**
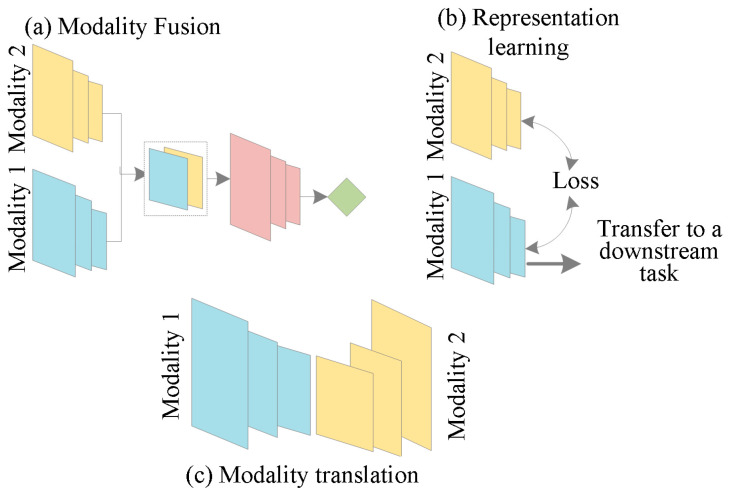
Classification of approaches using multiple modalities. Each approach is evaluated according to its utilization of modality combinations: (**a**) fusing the features of different modalities, (**b**) learning-based enhanced representation of different data types, and (**c**) translation of one type of data into another.

The classification of MMML in radiology encompasses several key concepts, among which modality fusion, cross-modality retrieval, and representation learning are predominant. Understanding these concepts is crucial for appreciating the intricacies of MMML. The published papers were classified based on methodology. This is predicated on the neural network’s “sections”, which connect every modality, as seen in Figure 3. In addition, a comprehensive collection of publicly accessible datasets in each modality category is also provided to assist researchers in finding a convenient starting point.

#### 3.2.1. Modality Fusion

Modality fusion refers to integrating data from multiple modalities to create a comprehensive model or representation. In radiology, this often means combining imaging and non-imaging data [65].

According to [16], fusion techniques may be classified as early, joint, or late fusion based on when features are fused within the neural network.

#### 3.2.2. Representation Learning

Representational learning in MMML refers to techniques used to automatically identify and learn the most effective ways of representing and integrating data for analysis. These approaches focus on acquiring enhanced feature depictions using information from several modalities. What is known regarding this problem encompasses methodologies such as co-learning, latent-space alignment, weakly supervised learning, and self-supervised learning [11]. This group includes studies integrating imaging and non-imaging data beyond simple feature fusion. It is beneficial when there is a lot of unlabeled multimodal data and a limited amount of labeled data.

#### 3.2.3. Cross-Modality Retrieval

Cross-modality retrieval in the context of MMML involves converting or interpreting information from one modality (radiology reports) to another (images), enhancing the understanding of a given medical scenario. This process is crucial when the direct comparison or integration of different data types is required for accurate diagnosis or treatment planning. This task is challenging since the neural networks are expected to acquire complex relationships between an image and the associated supportive information.

#### 3.2.4. Databases

Finally, a collection of open-access datasets is provided that includes radiological imagery and data from other modalities. This comprehensive analysis of the methods, along with data, offers the reader a complete understanding of the integration of modalities.

### 3.3. Target Readers

The target readership for this review on MMML in radiology is primarily AI-informed radiologists and computer scientists. This delineation is crucial as the content straddles the interdisciplinary nexus of advanced radiological practices and cutting-edge computational technologies.

AI-informed radiologists are radiologists with an interest or background in artificial intelligence (AI) and its applications in medical imaging. This review aims to provide these professionals with comprehensive insights into the latest advancements and applications of MMML in their field. The emphasis on categorizing data depending on its modality could be valuable to radiologists, since it could provide them with a comprehensive picture of the datasets and benchmarks. In addition, this review will undoubtedly assist in identifying limitations in data accessibility from a multimodal standpoint, hence promoting the creation of new databases available to the public.

Computer scientists specializing in medical imaging or AI represent this review’s second primary audience. The second type of classification, specifically designed for computer scientists, will provide a comprehensive review of the algorithmic advancements, focusing on the technical elements of learning across different modalities. In addition, this review’s emphasis on showcasing the therapeutic problems that MMML has been used to tackle will streamline the process of algorithmic improvement using the latest advancements in the field.

### 3.4. Study Selection

**Figure 4:**
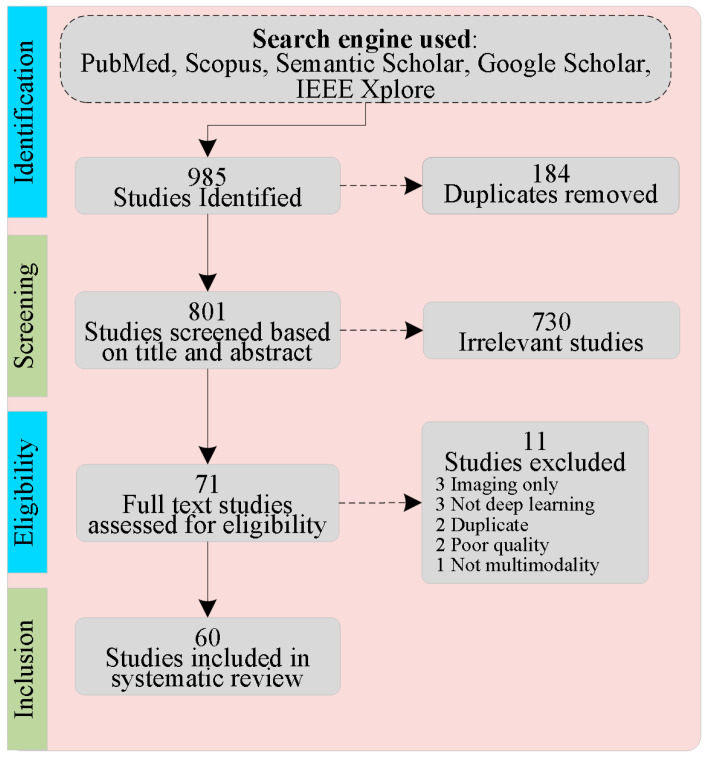
PRISMA flow diagram of the literature screening process. This flow chart delineates a systematic approach for searching and choosing studies for consideration in the review. It outlines the number of documents found, included, and eliminated at each phase of the searching and choosing process, from the initial database search to the final approved papers.

Comprehensive research was conducted across many databases, which include Semantic Scholar, Google Scholar, PubMed, IEEE Xplore Digital Library, and the ACM Digital Library. All the papers included in this survey are limited to the medical domain. Peer-reviewed journal articles and conference papers were selected, ensuring the relevance and recency of information. Articles not directly related to the application of MMDL in radiology or those lacking empirical evidence were excluded. This search strategy ensured a comprehensive and up-to-date literature collection, forming this review’s foundation. Figure 4 depicts a PRISMA flow diagram used to monitor the number of publications at each step of the screening phase. Following the processes outlined, 60 publications were selected on MMDL in radiology applications using fusion, representation learning, and cross-modality translation.

## 4. Multimodal Machine Learning in Radiology

Unlike many previous reviews organizing multimodal medical AI research by disease domain, this review focuses only on multimodal machine learning (MMML) in radiology. It utilizes a modality-based and methodology-driven classification paradigm, as described in Section 3. This methodology represents the framework of radiological data processes, whereby imaging is often combined with other non-imaging data types across several tasks, such as clinical reports, laboratory results, and vital signs. Figure 1 provides a streamlined design that links integrated data modalities to clinical goals (e.g., diagnosis, prognosis, and report production) using fusion, representation learning, and cross-modality conversion approaches. This structure ensures that the technical depth of MMML models is preserved while remaining clinically grounded and accessible to a broad audience.

### 4.1. Combination of Medical Imaging with Text Data for Diagnostic Precision

Integrating imaging and text data is a cornerstone in advancing MMDL applications in radiology. This combination enhances diagnostic accuracy and offers a more holistic patient assessment. An overview is provided in this section to analyze and manipulate images and text. The text is usually expressed in natural language but focuses on the medical field, such as radiology reports. Typical tasks in this combination include the classification of images enhanced by text data, the translation of modalities for report development, the retrieval of images, and the generation of captions.

#### 4.1.1. Fusion

Fusing imaging data with text data like patient notes and clinical history has led to significant advancements. Before the widespread use of the transformer approach, Wang et al. [66] introduced TieNet, a CNN+RNN design for text–image integration. TieNet can retrieve distinctive text image representations from sets of medical reports and chest X-rays (CXR) and can be trained end-to-end. The image processing system is based on the ResNet [67] architecture, while the text is encoded using an LSTM [68]. The performed tests demonstrate that using multimodal inputs instead of unimodal inputs leads to an enhanced AUC in multi-label disease classification. With the introduction of fully attention-based designs in natural language processing (NLP) [69] and their adaptation to computer vision [70], it is possible to create an integrated framework for processing images and text simultaneously. Li et al. [71] compared transformer-based image-and-language systems in the medical field. To be more specific, they use PixelBert [72], UNITER [73], VisualBERT [74], and LXMERT [75] models that have been trained using paired CXR and medical reports. In their research, the transformer-based joint fusion strategy outperforms TieNet and unimodal text-only designs, such as ClinicalBERT [76], BERT [77], and TieNet. VisualBERT had the highest performance among multimodal designs, with an AUC of 98.7%. In contrast, the ClinicalBERT model achieved the highest performance among the unimodal models, with an AUC value of 97.4%.

The work in [76] introduces a framework named MIFTP (Multi-level Independent Fusion with improved Twin Pyramid) for classifying multimodal medical image–text data, with a special focus on multilabel CXR images. The authors provide a multi-level, independent fusion method to integrate high-level and low-level image information with text features, tackling the problems of mutual interference and semantic deficiencies in conventional fusion techniques. The approach employs a Twin Pyramid module to augment semantic information in low-level image characteristics, enhancing their fusion efficacy with text features. The method surpasses other techniques on the MIMIC-CXR dataset in accurately identifying specific symptoms for CXR classification with Micro AUROC and accuracy scores of 0.706 and 0.915, respectively.

Shetty et al. [77] examine a deep learning architecture for detecting pulmonary defects by combining data from CXR with clinical text reports. The authors proposed two unimodal subnetworks (Unimodal Medical Text Embedding Subnetwork (UM-TES) and Unimodal Medical Visual Encoding Subnetwork (UMVES)) to extract the textual and imaging characteristics from the clinical text reports and CXR, respectively, and two multimodal designs, Compact Bilinear Pooling (CBP) and Deep Hadamard Product (DHP), which focus on the capture of inter-modal interactions between image and text information. These fusion methods complement data from both modalities to increase diagnosis accuracy over unimodal methods. The models were evaluated using datasets from Indiana University, demonstrating higher performance for multimodal approaches than unimodal ones.

Two multimodal approaches were investigated in [78] using early and late fusion. The early fusion framework combines image–text data at the input stage, using cross-attention to enhance relational comprehension. In contrast, late fusion integrates retrieved text and image features to improve diagnostic accuracy. They used BERT for sequence analysis and ResNet50 for image classification. Through extensive evaluation, late fusion showed superiority, achieving an outstanding accuracy of 94.2% and an exact match ratio of 51.2%, surpassing unimodal and early fusion approaches using the MIMIC-CXR dataset.

The study [79] introduces the medical cross-attention vision–language model (Medical X-VL), aimed at tackling the problems of merging visual and textual information in the medical domain. Medical X-VL integrates self-supervised unimodal classifiers with a fusion encoder, using momentum distillation and sentence-wise contrastive learning to enhance the alignment of medical reports with images. The model employs a vision transformer (ViT) for image processing and a self-supervised CXR-BERT approach for textual processing. A cross-attention-based fusion encoder merges the individual elements. The deep learning network utilizes contrastive learning and momentum distillation to properly synchronize image and textual input. The Medical X-VL surpasses previous models in error identification and correction within medical imaging using the MIMIC-CXR dataset.

Deng et al. [80] introduced a model training technique named Diagnostic Report Supervised Contrastive Learning (DRSCL) for the diagnosis of COVID-19, using a mix of convolutional neural networks (CNNs) and transformers for integrating images and text. The framework integrates textual data from medical reports during pre-training to enhance its generalization and interpretability. The model utilizes chest CT scans and diagnostic reports to improve feature learning and implements a contrastive learning approach to integrate similar text or image features, enhancing feature learning and ensuring convergence. It further incorporates a hierarchical fine-tuning method and has shown enhanced classification performance by joint fusion on a custom chest CT-text dataset.

The integration of medical imaging and textual data, shown by chest X-rays with radiology reports, reflects the clinical process in which visual observations are contextualized via narrative analysis. Techniques such as TieNet [66] and VisualBERT [74] exhibit early and joint fusion tactics by amalgamating CNN-derived image features with LSTM or transformer-based textual representations, attaining AUCs of up to 0.989 and 98.7% on MIMIC-CXR, respectively. These methodologies enhance multi-label disease categorization and replicate how radiologists integrate information across modalities. Although fusion improves interpretability and robustness, difficulties remain in synchronizing visual and textual data, owing to report variability and possible noise. These strategies replicate how doctors use descriptive and visual information to mitigate diagnostic uncertainty in tasks like pneumonia or cardiomegaly identification.

#### 4.1.2. Representation Learning

Chauhan et al. [81] developed a deep network trained on CXR and their corresponding expert reports using a semi-supervised approach. This training aims to accurately evaluate the degree of pulmonary edema. The images were analyzed via residual blocks, while BERT translated the radiological reports. The model’s training process minimizes the objective function, incorporating a joint embedding loss. This promotes the closer representations of matched pairs compared to mismatched pairings to two cross-entropy losses. The labels and the associated data come from the reports and the MIMIC-CXR dataset, respectively. The research demonstrates that joint embeddings outperform pre-trained image encoders with a macro-F1 measure of 0.51 against 0.43 for supervised image training.

The study in [82] presents a transformer (ViT-B/16)-based medical visual representation learning model that integrates contrastive learning and masked autoencoding into a single strategy. The proposed contrastive masked image–text model (CMITM) seeks to use the advantages of both methodologies to improve representation learning from paired medical images and textual data. The approach performs cross-modal contrastive learning between masked medical images and textual reports, using a representation decoder to retrieve misaligned information. Furthermore, it requires a masked image to restore the original image and masked data in the textual reports, enhancing masked autoencoding. Assessment across four classification datasets demonstrates continuous improvement with AUC scores of 0.86, 89.2, and 93.4, and accuracy of 95.3 when trained with 100 percent data.

ConVIRT architecture proposes to acquire visual representations from associated images and text in an unsupervised manner [83]. This approach compares text and image representations from text and image encoding pipelines. The BERT encoder condenses the text into a representation, while a ResNet-50 structure was used in the image encoding pipeline. Minimizing the bidirectional contrastive objective function enhances the alignment of genuine image–text pairings compared to random pairings. The weights of the image encoder are used in the empirical analysis as an initialization for the downstream tasks. Specifically, they complete an image–image and text–image retrieval challenge, along with four medical image classification tasks. ConVIRT outperforms other successful in-domain initiation methods that also make use of paired image–text data, such as ImageNet pre-training. Furthermore, ConVIRT uses far fewer data while still achieving comparison with them. In addition to investigating the efficiency of multimodal pre-training, they give tangible proof of the advantage of ConVIRT compared to single-modal unsupervised image representational learning strategies.

Huang et al. [48] propose GLoRIA, a contrastive learning model using attention to learn local and global representations of images. Inspired by the discovery that diseases typically represent tiny areas of the medical images, they learn both local and global characteristics together. The loss function comprises local contrastive loss to align the attention-weighted image and text representations and global contrastive loss to align text and image representations of a positive pair. A BERT architecture is used to encode the reports, and a ResNet-50 architecture is used to encode the medical images. The investigations used the datasets of RSNA Pneumonia, CheXpert, and SIIM Pneumothorax1. GloRIA delivers state-of-the-art performance in image–text retrieval and image classification tasks by being more label-efficient. GloRIA delivers the most effective results in image classification and image–text retrieval tasks by being more label-efficient.

Müller et al. [84] introduce the LoVT pre-training strategy, which enhances ConVIRT by including the learning of localized elements in both vision and text. LoVT utilizes ResNet-50, which encodes the image, and BERT to encode the text. Unlike [48], text encoding operates at the sentence level rather than that of individual words. Moreover, a local contrastive loss is used by an attention-based aligning approach to compute cross-modal predictions that are aligned with the unimodal predictions. The local and global contrastive losses are reduced to acquire knowledge about their model. The extensive tests use publicly accessible CXR databases, including 18 specific downstream tasks, from object recognition to semantic segmentation. The self-supervised image–text approaches, such LoVT or ConVIRT, outperform their unimodal (self-supervised) counterparts in fifteen of eighteen cases. LoVT achieves the highest scores in eleven of eighteen downstream tests. Their research demonstrates that multimodal pre-training is more effective than unimodal pre-training and offers more proof of label efficiency.

Using combined data, such as paired and unpaired text reports and images, a transformer-based text–image pre-training architecture is proposed in [85]. This framework is designed to acquire the representations of both modalities mutually. In the transformer design, both encoders exchange weights, which serves as a source of inspiration for the encoding of each modality. They present two attention-based modules that may be positioned between the encoder and the decoder to describe the relationship between the text and images. The UNIT WithOut Cross Fusion (UWOX) unit does not need text and image data for training since it uses self-attention when sharing weights. On the other hand, the UNIfied Transformer (UNIT) module requires both modalities for training and achieves cross-modality cross-attention. The framework is acquired by minimalizing the loss in predicting masked words, masked patches, and matching pairs. The network undergoes training using the NIH14 and MIMIC CXR databases, whereas the OpenI-CXR database is used for external authentication. In classification, retrieval, and image synthesis tasks, they outperform unimodal transformer models, yielding superior results.

Table 1 provides details of the fusion and representation learning studies utilizing imaging and text modalities included in this review. Models for representation learning, including GLoRIA [48], ConVIRT [83], and CMITM [82], utilize contrastive learning to align medical images with corresponding text, facilitating strong joint embeddings for subsequent tasks such as classification, retrieval, and report generation. These models undergo pre-training on extensive, paired datasets to address the challenge of limited labels, employing attention mechanisms and transformers to concentrate on clinically pertinent regions and phrases (e.g., “pleural effusion”). GLoRIA enhances interpretability through localized alignment, whereas ConVIRT attains robust performance (92.7% AUC on CheXpert) by utilizing the structure of concurrent image–text data. The effectiveness is limited by the variability in clinical narratives and the inconsistent alignment between modalities. These methods illustrate a radiologist’s strategy for constructing context through the integration of image features and textual interpretations.

**Table 1:** Performance overview of fusion and representation learning works utilizing imaging and text modalities comprised in this review (MM stands for multimodal).

Reference	Framework	Dataset	Image Modality	Text Modality	Fusion/Representation	Metric	MM Performance
[66]	TieNet	ChestX-ray14	CXR	Text reports	Joint fusion	AUC	0.989
[71]	VisualBERT LXMERT UNITER PixelBERT	MIMIC-CXR	CXR	Text reports	Joint fusion	AUC AUC AUC AUC	0.987 0.984 0.986 0.974
[76]	MIFTP	MIMIC-CXR	CXR	Text reports	Joint fusion	Micro AUROC accuracy	0.706 0.915
[77]	CBP-MMFN DHP-MMFN	IU-X-ray	CXR	Text reports	Joint fusion	AUROC accuracy, AUROC accuracy	0.987 0.988 0.984 0.972
[78]	ResNet50 BERT	MIMIC-CXR	CXR	Text reports	Early fusion Late fusion	Accuracy Accuracy	0.942 0.914
[79]	Medical X-VL	MIMIC-CXR	CXR	Text reports	Joint fusion	AUC F1 score	0.855 0.516
[80]	DRSCL	Private	CT	Text reports	Joint fusion	Accuracy F1 score	0.901 0.903
[81]	Resnet, BERT	MIMIC-CXR	CXR	Text reports	Ranking-based	Macro-F1	0.51
[83]	ConVIRT	CheXpert	CXR	Text reports	Contrastive	AUC AUC Accuracy AUC	0.927 0.881 0.924 0.890
[48]	GLoRIA	CheXpert, RSNA Pneumonia, SIIM Pneumothorax1	CXR	Text reports	Contrastive	AUC AUC Dice	0.881 0.886 0.634
[84]	LoVT	MIMIC-CXR RSNA	CXR	Text reports	Contrastive	mAP fROC Dice Dice	0.181 0.621 0.512 0.441
[85]	UWOX	NIH14-CXR, MIMIC-CXR	CXR	Text reports	Masked (Word, Patch) prediction	AUC	0.763
[82]	CMITM, ViT-B/16	MIMIC-CXR NIH-CXR CheXpert RSNA	CXR	Text reports	Contrastive, masked autoencoding	AUC AUC AUC accuracy	0.860 0.892 0.934 0.953

#### 4.1.3. Cross-Modality Retrieval

The cross-modality retrieval between imaging and text data presents unique challenges, primarily due to the differing data structures and formats. Translation can be text-to-image or image-to-text. In this review, text-to-image involves image synthesis, while image-to-text involves challenges in medical report generation and visual question answering (VQA).

**Report generation:** The objective of report generation is to automatically produce descriptions from medical imagery. The training of the report generation process requires both medical images and clinical text reports, hence classifying it as a multimodal learning technique. BLEU, METEOR, and ROUGE-L scores are calculated between the actual reports and the created counterparts to assess the excellence of the produced text report. These measurements indicate the overlapping statistics between two text corpora.

Boag et al. [86] present a range of methods for generating text from images. These methods include employing n-gram models conditioned on images, retrieving reports based on the closest image using a query image (CNN), and simultaneously learning from both text and image utilizing RNN-CNN architectures. For the most part, this lays the foundation for the efforts made in this field. Typically, RNN-based production is divided into word and sentence decoders [87]. The sentence decoder produces sentence topics from the image representations. On the other hand, the word generator generates word sequences by adhering to the image representations and using the generated topics. Advancements in this area include improving the representations of the query image, such as employing triplet and matching loss functions [85] or utilizing a knowledge graph [88]. The article [88] introduced a method to enhance radiology report generation by incorporating a graph-embedding module based on a graph CNN. The study proposed a deep learning model using CNN-RNN architectures and attention mechanisms by addressing the importance of accurate disease mentions in radiology reporting. Through experiments on the IU-RR dataset, the knowledge graph integration demonstrated superior performance in classification and report generation tasks, showcasing accuracy and clinical relevance advancements.

In recent studies, transformers have replaced RNN structures for language modeling, representing logical development. The research in [89] introduces a method for generating radiology reports using a memory-driven transformer. Their approach includes relations-based storage for storing essential data throughout the report generation procedure and utilizing memory-driven conditioned layer regularization in the transformer decoder to implicitly model and remember comparable patterns in medical reports throughout the generation. The VMEKNet framework was presented by Chen et al. [90], which integrates the transformer with external knowledge and visual memory, delivering enhanced efficiency for both quantitative and qualitative assessments and medical diagnosis. You et al. [91] proposed the align transformer structure, which consists of AHA and MGT modules. The AHA unit detects disease labels from images and aligns visual areas to improve abnormal region representation and reduce data bias. The MGT module then utilizes these capabilities inside a transformer architecture to provide complete medical reports. It is important to note that in the majority of works mentioned above, it is necessary to have paired images and reports. In [92], the demand for such a requirement is removed by utilizing a modality-free feature learning technique enhanced by a pre-existent information graph. The Knowledge Graph Auto-Encoder (KGAE) processes separate groups of reports and images during training. It comprises a pre-assembled knowledge graph, a knowledge-based encoder, and a knowledge-based decoder.

KERP [93] employs retrieval-based generation, wherein text samples are retrieved using image encoding and subsequently rephrased to produce the concluding description. All three stages of KERP, encoding, retrieving, and rephrasing, rely on graph-based methods. In the context of retrieving reports based on images, ref. [94] employs a CNN to produce core labels, which are the primary labels assigned to the images, as well as fine-finding labels, which are structural sets of labels produced by analyzing the reports. The ultimate report is generated by retrieving a “nearest” report from the repository and then post-processing it. The retrieval-based technique called CXR-RePaiR (Contrastive X-ray-Report Pair Retrieval) proposed in [95] utilizes characterizations acquired through contrastive learning to calculate similarity for report generation.

Tanwani et al.’s RepsNet model [96] integrates the concept of self-supervised contrastive aligning. RepsNet comprises an encoder–decoder architecture whereby the encoder aligns images with natural language descriptions using contrastive learning, and the decoder forecasts answers by conditioning on encoded images and the previous context of descriptions obtained via closest neighbor search. Certain studies have concentrated on enhancing the accuracy and comprehensiveness of produced reports using rewards systems. Miura et al. [97] proposed a framework that incorporates the reward system into reinforcement training, yielding substantial improvements in clinical routine, which was subsequently enhanced by Delbrouck et al. [98], resulting in a 14.2% increase in factual accuracy and a 25.3% improvement in completeness.

Wang et al. [99] address developments in the creation of medical reports from X-ray scans via the integration of Large Language Models (LLMs) with ViT models. It highlights the difficulty of obtaining more efficient info to improve LLM efficiency and the computational complexities imposed by ViTs. The research presents a context-guided efficient framework that employs the Mamba vision backbone with linear complexity to enhance outcomes equivalent to robust transformer frameworks. It incorporates context retrieval during training to improve feature representation and discriminative learning. The suggested technique creates high-quality medical reports by integrating vision tokens, contextual data, and swift statements. Comprehensive testing on three X-ray report datasets confirms the efficacy of the suggested method.

The task of image captioning is derived from the task of generating reports. This assignment is perhaps easier because it places less emphasis on the output text’s need to be natural, smooth, and clinically accurate. Nevertheless, a limited number of studies [100] tackle this objective, and they are based on the widely used CNN-RNN architecture. Supplementary Table S1 details the literature used in this paper for report generation using medical imaging and text data.

**Visual question answering:** Visual question answering (VQA) is the process of answering questions based on visual information. The majority of the methods in this field focus on the ImageCLEF dataset. Typically, VQA strategies employ a framework consisting of two encoders and a decoder. The encoders are used for querying images and text, subsequently preceded by a text decoder. It is essential to know that VQA requires less natural and clinically correct text language than report creation, similar to image captioning.

Table 2 provides a summary of the literature using MMDL implementation in VQA. Image-CLEF has been conducting an annual clinical VQA competition since 2018 to assess and grade the efficacy of competing methods. The prominent VQA databases in the healthcare field are VQA-MED-2020 [101], VQA-MED-2019 [102], and VQA-MED-2018 [103], which were introduced via competition challenges. These files include radiological images and their associated question–answer pairs.

Liu et al. [104] suggested a two-branch model that utilizes both ResNet-152 and VGG-16 to retrieve sequential and spatial attributes, improving the semantic comprehension of images by retrieving image feature similarities. RNNs, including Gated Recurrent Unit (GRU) and Long Short-Term Memory (LSTM), are frequently employed to retrieve question attributes. Furthermore, BERT-based designs [105] are increasingly implemented for textual feature retrieval. Models from the broad area of VQA, including Multimodal Factorized Bilinear (MFB) [106], Bilinear Attention Networks (BANs), Stacked Attention Networks (SANs), and Multimodal Factorized High-order (MFH) [107], are commonly used in multimodal fusion. MFB was used as a feature fusion technique by Sherma et al. [108] to create an attention-based framework that decreases complexity and improves performance. The contrastive pre-training and representation process (CPRD), a pre-training method introduced by Liu et al. [109], performs well and successfully handles the problem of insufficient MED-VQA data. The multiple Meta-model Quantifying (MMQ) framework proposed by Do et al. [110] attains exceptional accuracy via metadata. According to recent developments, the most effective systems are BERT and attention-based systems, which are anticipated to dominate the field of VQA algorithms in the future.

Chen et al. [111] present a self-supervised learning approach using multimodal masked autoencoders for pre-training in medical vision and language. The method acquires cross-modal domain knowledge by reproducing absent tokens and pixels from randomly masked texts and images. Three principal designs implement varying masking ratios corresponding to the disparate information densities of language and vision, using textual and visual features from distinct layers for reconstruction and creating different structures for vision and language decoders, including transformers for vision and MLP for language. The study by Chen et al. [112] proposes a systematic approach to improve medical vision and language pre-training by structured medical knowledge. Their approach aligns vision and language encoder representations through knowledge, injects knowledge into a multimodal fusion architecture, and guides it to emphasize important information in text and images through knowledge-induced pretext tasks. This approach enhances the model’s reasoning capabilities.

Eslami et al. [113] investigated the efficacy of the CLIP method for Medical VQA (MedVQA). They presented a specialized variant of CLIP called PubMedCLIP, fine-tuned using PubMed articles and trained on medical images from many anatomical organs. Experiments conducted on two MedVQA benchmark datasets demonstrate that PubMedCLIP surpasses current methodologies, enhancing overall accuracy by as much as 3% relative to Model-Agnostic Meta-Learning (MAML) networks pre-trained only on visual data. Gong et al. [111] provide a self-supervised mechanism according to the “pretrain and finetune” approach for dealing with medical VQA challenges as generative operations.

**Table 2:** Performance in studies utilizing MMML for VQA using medical imaging and text data. ACC, O.E., and C.E stand for accuracy, open-ended, and close-ended questions, respectively.

Reference	Framework	Dataset	Text Modality	Image Modality	Metric	MM Performance
[114]	BERT, Vision transformer	VQA-RAD, SLAKE, ImageCLEF 2019	Medical questions	Radiology scans	ACC	0.785 0.835 0.833
[112]	BERT Vision transformer	VQA-RAD, SLACK, ImageCLEF 2019	Clinical questions	Radiology scans	ACC	0.803 0.856 0.791
[110]	SAN/BAN, MMQ, LSTM	VQA-RAD PathVQA	Clinical questions	Radiology scans	ACC	0.488 0.670
[113]	QCR, MEVF, CLIP	VQA-RAD SLAKE	Clinical questions	Radiology scans	ACC	0.801 0.721
[111]	LSTM, CNN	VQA-RAD	Clinical questions	Radiology scans	ACC	0.732
[109]	CPRD, BAN, LSTM, SLAKE	VQA-RAD	Clinical questions	Radiology scans	ACC	0.678
[104]	CNN, transformer	VQA-RAD ImageCLEF (2018, 2019)	Clinical questions	Radiology scans	BLEU ACC ACC	0.162 0.654 0.727
[115]	GRU, CDAE, MAML	PathVQA VQA-RAD	Clinical questions	Radiology scans	ACC	0.504 0.733
[108]	MFB, BERT, CNN	ImageCLEF 2019	Clinical questions	Radiology scans	ACC, AUC-ROC AUC-PRC	0.636 0.800 0.618
[96]	BAN, BERT, CNN	VQA-RAD	Clinical questions	Radiology scans	Accuracy	0.804

This approach comprises Masked Language Modeling, Masked Image Modeling, Image Text Matching, and Image Text Contrastive Learning (M2I2) as pre-training goals to acquire unimodal and multimodal feature representations from an image caption dataset. After that, these representations are effectively applied to subsequent medical VQA tasks. Their approach attains superior results on three medical VQA databases with improvements of 1.3%, 13.6%, and 1.1%.

**Image synthesis:** Image synthesis is a well-studied research theme in computer vision, owing to the introduction of generative adversarial networks (GANs). The created images can frequently enhance the training resources for a subsequent activity. Just like the class-conditioned synthesis of ImageNet samples, they can be used to supplement data for improved classifier training. However, in radiology, conditioned image synthesis has received somewhat less attention. This may be due to higher criteria for utilizing the generated image in subsequent activity.

Kim et al. [116] proposed an approach for text-conditional MRI synthesis, addressing multimodality challenges. It incorporates a pre-trained language model, a diffusion-based image-generating module, and a structural binary mask denoising network. The system can create MR images matched with medical text prompting and understand text-based cross-attention maps. A model for creating high-quality medical images with full anatomical information was developed by [117]. The method combines advanced NLP with image generation to precisely align text prompts with anatomical details. An anatomy–pathology prompting module provides descriptive prompts, and a fine-grained alignment-based generation mechanism employs a visual codebook to match the prompts with critical visual patches to improve image accuracy. Che Liu et al. [118] investigated synthetic images derived from radiology reports for vision–language pre-training (VLP) as a substitute for real images. Three sophisticated VLP algorithms were trained only on synthetic data, and their efficiency was assessed in image classification, segmentation, and object detection. The results indicate that synthetic data perform comparably to or surpasses real images. Reference [119] is a study that focuses on generating images (CXRs) from radiology records using a progressive GAN.

Numerous cross-modal retrieval problems depend on aligning text and image characteristics via contrastive learning. This procedure encompasses local and global feature alignment in conjunction with attention processes. In [114], Chen et al. devised self-supervised multimodal masked autoencoders, attaining remarkable results in text-to-image retrieval using the ROCO database. Maleki et al. [120] introduced LILE, a dual attention framework that employs transformers and an extra self-attention loss term to improve interior characteristics for image recovery using the ARCH collection.

Supplementary Table S2 details the studies used in this paper for report generation using medical imaging and text data. Cross-modal retrieval connects medical imaging and text by facilitating automated report preparation, question answering, and image captioning. Models like KERP [93] and RepsNet [96] synchronize visual characteristics with diagnostic terms using graph-based or contrastive learning, attaining a BLEU-4 score of 0.58 on IU X-ray. Transformer-based designs enhance retrieval and generation quality by modeling attention between image areas and textual elements. Despite these advancements, assessment measures often inadequately reflect clinical accuracy, and the resultant text may exclude or mislead essential facts. These models emulate the documentation practices of radiologists and address diagnostic enquiries to enhance clinical communication and alleviate the reporting workload.

#### 4.1.4. Specific Datasets Used in This Domain

Supplementary Table S3 provides a list of freely accessible multimodal databases. The MIMIC-CXR collection is a significant collection of images and text. It consists of 227,835 chest X-rays from 65,379 individuals, and semi-structured text reports [121]. In the JPEG version of this dataset called MIMIC-CXR-JPG [122], the studies are categorized into fourteen structural labels utilizing two rules-based NLP techniques. Many extensive collections of chest X-rays are available, including the Indiana Chest X-ray (IU X-ray) dataset [120]. This collection contains 3996 radiology reports and 8121 accompanying X-ray scans with different views. The PADCHEST database [123], sourced from the San Juan Hospital, is a substantial collection of healthcare images. It comprises 160,000 images collected from 67,000 individuals. These images are labeled for 174 radiological results, 19 various diagnoses, and 104 anatomic areas. The labels are organized systematically according to the Unified Medical Language System. PADCHEST also offers complimentary radiological reports in Spanish. The succeeding sections address the common use of these collections in studies related to multi-label image classification, report production, and image recovery. The ImageCLEF [124] is another widely used data source for studying the combination of image and text genres. These challenges primarily focus on language-free image labeling, multimodal knowledge retrieving, and image recovery. The data mainly comprise radiology images obtained by searching PubMed publications. The dataset includes image captions and encompasses various medical imaging such as X-rays, MRI, CT scans, and angiography. The RadGraph [125] dataset contains radiology reports, annotated entities, and relations for each report. The training set consists of 500 MIMIC-CXR radiology reports, while the test set consists of 50 MIMIC-CXR and 50 CheXpert reports, which are independently annotated by two board-certified radiologists.

The COV-CTR [126] consists of 728 images (379 for non-COVID-19 and 349 for COVID-19) collected from published papers and their corresponding paired reports. The COVID-19 CT [127] dataset, which contains 368 medical findings in Chinese and 1104 chest CT scans, was constructed. The CANDID-PTX database [128] is also available online. It includes 19,237 chest X-rays together with their related reports from radiologists. CANDID-PTX has line marks for chest tubes, bounding boxes for acute rib fractures, area annotations, and visual segmentation annotations for pneumothorax. CheXpert [129] contains 191,229 CXR images for multi-label classification, i.e., pleural effusion, edema, consolidation, cardiomegaly, and atelectasis. NIH CXR [130] comprises 112,120 CXR scans with fourteen disease labels. Each CXR can be associated with multiple diseases. In total, 29,684 CXRs, for a binary classification problem when differentiating between pneumonia and normal X-rays, are included in the RSNA pneumonia dataset [131]. The MedICaT [132] and the ROCO [133] collections are also retrieved similarly, specifically focusing on figures and captions. These databases are utilized for activities such as image labeling and concept labeling. VQA-MED-2020 contains 4500 X-ray scans and 4500 QA pairs [101]. Moreover, VQA-RAD [134] and Path VQA [135] are also available. SLAKE, a bilingual dataset by Liu et al. [136], used for medical VQA, comprises semantic labels, structural medical knowledge, and additional modalities and body parts, and is available for public use. A summary of freely accessible datasets is provided in Supplementary Table S3.

#### 4.1.5. Author’s Insights

Interestingly, there is still a scarcity of work that addresses image and text fusion. This is anticipated, as most databases, like PADCHEST and MIMIC-CXR-JPG, depend on extracting labels from the related radiological reports via rule-based methods. This industry would be more profitable if the labeling were created by humans or by a process that does not rely on any modality. From the viewpoint of representational learning, it is observed that BERT [137] serves as a significant influence on several NLP-related methods ([84]). This notion is supported by the widespread use of transformer topologies in addressing diverse problems. Compared to supervised unimodal approaches, representation learning that combines image and text modalities performs better in several downstream tasks. The modality translation from image to text is the sub-domain examined. The present cutting edge in report creation and image captioning entails using transformer topologies for processing both images and text [89]. To further promote study of this combination of modalities, there is a substantial amount of publicly available data. However, according to the data shown in Supplementary Tables S1 and S2, most of these databases concentrate on CXR. Diversifying this area may result in a more intriguing investigation into an existing productive sector.

The integration of imaging and text data in these applications highlights the potential of MMML systems to enhance clinical decision-making. Continued innovations in fusion and representation learning are driving these models toward practical deployment in real-world settings.

### 4.2. Combination of Medical Imaging with Structured Data for Diagnostic Precision

This part examines the integration of structured data with medical images. Structured data often obtained from electronic healthcare reports (EHR) are commonly displayed in a spreadsheet format. Columns represent discrete and continuous measurements, while rows reflect the data’s underlying chronological ordering. If one does not exist, the spreadsheet’s rows represent the various patients (cross-sectional data). A time-based arrangement indicates that the rows reflect points in time. Rows may show a solo point over time (time series data) or a point in time together with an individual’s identity (longitudinal data). Possible variables can consist of details about demographics, such as the patient’s gender, age, height, and weight, as well as health indicators, like blood pressure, heart rate, and temperature, or additional laboratory investigations or evaluations.

#### 4.2.1. Fusion

When combining medical imaging and diagnostic data in tabular form, a comprehensive study [16] is recommended for reference. The study provides implementation directions for evaluating the performance disparities between early, intermediate, and late fusion methods. A list of publications released after reference [16] is provided here.

The authors of [17] developed early, joint, and late multimodal fusion frameworks to detect pulmonary embolism. They employed a private dataset of HER and CT images to train seven multimodal and two unimodal fusion models. The PENet [138] structure served as the computer vision base, while a feed-forward network encoded the structured information. With an AUC of 0.947, the late fusion outperformed the text-only and image-only models, which had respective AUCs of 0.911 and 0.791. In addition to introducing the RadFusion dataset, Zhou et al. [139] also investigated both multimodal and unimodal frameworks to evaluate the performance of pulmonary embolism identification. The PENet model is designed to process the CT image data, while the ElasticNet model is specifically designed for EHR tabular data. The multimodal framework is a late fusion strategy created by combining the predicted outcomes of unimodal models using mean pooling. The multimodal late fusion framework achieves a higher AUC of 0.946 than the ElasticNet network, with an AUC of 0.922 and the PENet network with an AUC of 0.796.

In their historical investigation for the classification of breast cancer, researchers used multimodal fusion models comprising two joint fusion frameworks and one late fusion architecture [140]. The private imagery and tabulated data consist of DCE-MRI scans with eighteen corresponding medical EHR measurements, such as mammographic breast density, clinical indication, and demographic information. The DCE-MRI scans were encoded using a ResNet-50 to create a 2D maximum-intensity projection. The tabular data are processed using an FFD (feed-forward) network. The fusion models consistently outperform their unimodal counterparts, both the image-only (AUC: 0.849) and text-only (AUC: 0.807) models, in every single experiment. The joint fusion technique is the most effective of all the fusion designs. Specifically, the joint fusion approach that utilizes learned images and tabular encoders performs most effectively. Sun et al. [141] proposed a framework based on an encoder–decoder structure that fuses MRI and EHR data to enhance the risk prediction of cerebral degeneration. It utilizes spatial–temporal and cross-attention techniques to efficiently capture intra-modal and inter-modal relationships. The decoder module utilizes a disease-centric methodology and a multi-head attention mechanism to extract essential disease features. This approach exhibits remarkable accuracy, attaining 0.859 for Alzheimer’s disease and 0.899 for diabetic mellitus on the Alzheimer’s Disease Neuroimaging Initiative (ADNI) database. The multimodal fusion approach surpasses MRI-only and EHR-only models, providing enhanced predictive performance.

In [142], the authors presented a model named Multimodal3DSiameseNet [143] designed for predicting cognitive decline for individuals with neurodegenerative disorders by utilizing multimodal data, which encompasses brain MRIs, clinical assessments, and various risk factors. This model implements a joint fusion strategy, amalgamating features from diverse modalities and processing them through fully connected layers to leverage correlations. It proficiently delivers long-term predictions of cognitive decline from any pair of initial visits, irrespective of the time interval. Furthermore, on the Parkinson’s dataset (PPMI), the model attains an AUC of 0.81 following transfer learning, in contrast to 0.72 when trained from scratch. In [144], the authors devised a technique to forecast lymph node metastases in non-small cell lung cancer by combining CT images and genomic data. They tackled issues such as limited sample numbers and inadequate multimodal fusion by developing an attention-based fusion module for joint fusion and a bilinear fusion module utilizing Tucker decomposition for late fusion. Random forest was used for feature selection to remove redundant data in genetic information. This enhancement greatly improved multimodal efficiency. When evaluated on the NSCLC-Radiogenomics dataset, the model attained an accuracy of 0.968 and an AUC of 0.963, illustrating its efficacy in non-invasive prediction and endorsing precision medicine.

In [145], the authors developed and compared multimodal fusion models to automatically assess pulmonary embolism (PE) severity by combining CT imaging and clinical data. XGBoost [146] and TabNet [147] were used for clinical data, while SANet [148] was used for imaging data classification. Their best model uses a joint fusion approach with bilinear attention and TabNet, which is trained end-to-end. This model improved performance by up to 14%, achieving an AUC of 0.96, with a sensitivity of 90% and specificity of 94%, highlighting the effectiveness of multimodal data in assessing PE severity. In [149], the researchers published the Dynamic Affine Feature Map Transform (DAFT), a module for CNN that blends 3D brain MRI data with low-dimensional tabular data, including demographics and laboratory data, for the diagnosis of Alzheimer’s disease.

DAFT dynamically modifies feature maps according to this tabular data. It considerably exceeded the diagnostic capability and time-to-dementia prediction, with a mean balanced accuracy of 0.622 and a mean c-index of 0.748.

Duvieusart et al. [28] utilized fusion to link biomarkers retrieved from images with demographic information, vital signs, and lab findings. They explored the automatic diagnosis of cardiomegaly by creating classifiers utilizing multimodal data augmented with digital biomarkers retrieved from images: the cardiothoracic ratio (CTR) and the cardiopulmonary area ratio (CPAR). These biomarkers were extracted from segmentation and detection models and incorporated into a network trained on CXR and ICU data. The XGBoost [146] model attained an AUC of 0.810, utilizing the extracted biomarkers above the ResNet-50 model’s AUC of 0.768. The study concluded that fusion models using these biomarkers perform comparably to more complex models, with higher clinical explanations. In [150], the authors used single-modality and multimodality strategies to examine lung cancer detection. A ResNet18 network was used to categorize 3D CT nodule regions, with an AUC of 0.789, while a random forest method classified clinical data, with an AUC of 0.5241. They used intermediate and late multimodality fusion approaches using CT and clinical data. The best model combined clinical data with deep imaging via a fully connected layer, yielding an AUC of 0.8021 on the National Lung Screening Trial (NLST) dataset, showing how multimodal fusion may improve predicted accuracy compared to single-modality models. In [151], MRI scans and demographic data were combined using joint fusion and CNN to predict the response to neoadjuvant chemotherapy in breast cancer. This was accomplished by multiplying the intermediate imaging and non-imaging values channel by channel. They examined a subset of selected data from the I-SPY-1 TRIAL, in which 112 patients with stage 2 or 3 breast cancer were selected who were given the standard neoadjuvant chemotherapy. They obtained an AUC of 0.80 and an accuracy of 0.83. Table 3 provides the details of fusion strategies for imaging and structured non-imaging data included in this paper.

The integration of imaging with structured clinical data, including laboratory results, vital signs, and demographic information, illustrates the diagnostic complexity encountered in clinical practice, where clinicians synthesize various sources of evidence. Methods such as PENet-ElasticNet [17] integrate CT images with laboratory variables (e.g., D-dimer levels) to detect pulmonary embolism, attaining a 0.947 AUC in this case and surpassing unimodal models. Joint and late fusion strategies employed in DAFT [149] for Alzheimer’s prediction dynamically adjust imaging features in conjunction with demographic data. Multimodal models enhance risk stratification and classification performance; however, they encounter challenges associated with missing values, feature normalization, and data heterogeneity. These techniques mirror the integrative reasoning employed by physicians, who assess structured and visual cues to inform their decision-making processes.

#### 4.2.2. Representation Learning

The SimCLR [152]-based contrastive learning framework, developed by [153], proposes the multi-instance contrasting learning approach (MICLe) that integrates pathological knowledge into the generation of positive pairs. Unlike classical contrastive learning in computer vision, where positive pairings are created by applying two transforms to a single image, MICLe generates positive pairs by applying two transforms to potentially different images that exhibit similar pathologies (i.e., originating from a single person). The subsequent challenges include the classification of dermatological conditions and the classification of CXR images with multiple labels. The scientific study highlights the advantage of expanding the set of positive pairs by employing patient metadata. Furthermore, they provided additional information to support the effectiveness of multimodal pre-training and demonstrated that their MICLe pre-training beat the unimodal SimCLR pre-training.

In [154], the authors create MedAug, a technique that utilizes individual metadata to choose positive combinations. MedAug, which is incorporated into a MoCo [155,156]-oriented contrastive learning architecture, additionally demands that the images of positive pairs must come from an identical individual. To create positive matches, the researchers investigate selection strategies according to laterality and study number. The findings in the subsequent pleural effusion categorization challenge enhance the research completed by [153], as the research demonstrates that the metadata influence the outcome. Specifically, they revealed that selecting all images from the same patient negatively impacted the subsequent efficiency. On the other hand, using a selection criterion using study numbers yielded excellent results.

The research in [157] proposes a multimodal pre-training method for chest radiographs that integrates EHR data to improve representation quality. Employing a Masked Siamese Network (MSN) and assessing with three chest X-ray datasets (MIMIC-CXR, CheXpert, NIH-14) with two vision transformer (ViT) architectures (ViT-Tiny and ViT-Small), the approach demonstrates substantial improvements in representation quality. It attains a 2% improvement in AUROC compared to the standard MSN and a 5–8% enhancement over alternative baselines, with demographic variables playing a pivotal role in the efficiency amplification.

Another paper by Holland et al. [158] presents a strategy that uses medical metadata to tackle the challenges associated with conventional contrastive learning methods in medical images. This method integrates patient metadata, including identity, eye position, and time series data, to enhance inter-image contrastive relationships. The proposed model demonstrates superior performance compared to conventional contrastive approaches and a retinal image foundation model across multiple image-level tasks associated with age-related macular degeneration (AMD) by utilizing this metadata in the pre-training phase. The approach effectively incorporates the temporal dynamics of disease progression into its learning process, demonstrating advantages in both low-data and high-data contexts. The modular design facilitates rapid and economical testing to assess the benefits of incorporating existing metadata in contrastive pre-training. Utilizing all 10,000 labeled samples, their methodology exceeds all baseline performances, achieving a 0.85 AUC in the Southampton dataset and 0.88 AUC in the Moorfields dataset.

The study [159] presents an innovative approach for survival prediction in non-small cell lung cancer (NSCLC) patients by merging CT and PET scans with genetic data. The method entails acquiring representations for each modality using a self-supervised module and assuring the alignment of embeddings according to semantic commonalities across patients. A Cross-Patient Modality (CPM) module is used to identify connections between modalities for patients exhibiting analogous disease features. A multimodal patient embedding module (MPE) minimizes the distances among the learned embeddings within each patient. This facilitates a closer alignment of the representations derived from CT, PET, and genetic data. Experimental findings on an NSCLC dataset indicate that the suggested model achieved a C-index of 0.756, showing a 9% enhancement in predictive performance compared to unimodal approaches and a 3% advancement over multimodal methods.

In another study [160], the authors propose a novel approach using clinical information and imaging data in contrastive learning to improve model generalization and interpretability. They introduce Patient-aware Contrastive Learning (PaCL). This includes an inter-class separability objective (IeSO) and an intra-class diversity objective (IaDO). IeSO refines samples using rich clinical information, while IaDO maintains necessary diversity to avoid class collapse. They demonstrate PaCL’s effectiveness both theoretically through causal refinements and empirically in medical imaging tasks, showing that PaCL outperforms previous methods. The authors of [161] introduce an approach to contrastive representation learning by incorporating continuous metadata. Their unsupervised model, pre-trained on a 3D brain MRI dataset, matches or exceeds the performance of fully supervised CNNs on three classification tasks. An ablation study further confirms that leveraging metadata improves performance across all downstream tasks. Table 4 provides a detailed overview of representation learning papers using image and structured non-image data included in this paper.

Representation learning using structured data improves model generalizability and contextual relevance by integrating patient-specific variables such as age, sex, and pathologies. Frameworks like MedAug [154] and MICLe [153] enhance classification tasks by pre-training on enhanced or contrastively aligned data derived from the same patient. These models mitigate dataset bias and enhance ongoing disease monitoring, akin to how medical professionals analyze imaging in conjunction with developing patient records. Dual-branch networks encode structured and imaging inputs concurrently before fusion, facilitating the identification of cross-modal correlations. Nonetheless, constraints emerge when structured data are either scarce or poorly temporally linked with imaging investigations.

#### 4.2.3. Cross-Modality Retrieval

One may envision that the process of cross-modality retrieval between the images and structured data (such as tables) involves deducing electronic health information or clinical metadata based on an image or creating an image based on clinical metadata. The practical value of translating metadata into images is uncertain, indicating an overall scarcity of research in the area. The authors of [162], for instance, generate images by combining clinical metadata, such as scanner kind. Given the limited use of data fields for generating images based on conditions, it is uncertain whether labeling this effort as tabular-to-image translation is appropriate. The opposite approach (image-to-tabular) also exhibits identical attributes, with a considerable number of works that might be categorized as image-to-tabular translation, often inferring only a small amount of metadata (such as patient age estimations [163], genomics signatures [164], etc.), which makes them typical regression or classification systems.

Although less prevalent than text-based systems, cross-modal retrieval between imaging and structured data enhances precision medicine by correlating clinical characteristics with imaging patterns. In particular, 3D ResNet models utilize MRIs for estimating patient age and APOE4 genetic markers [141,142], while radiogenomic methods correlate CT radiomics with mutation status (e.g., EGFR) [144]. These technologies emulate a clinician’s endeavor to deduce health conditions from non-visual measures and diminish dependence on intrusive tests. Retrieval efficiency is constrained by the semantic disparity between structured inputs and visual attributes, as well as structured data’s failure to express diagnostic details. However, this method encourages clinical decision-making using analogical thinking.

#### 4.2.4. Datasets

The Alzheimer’s Disease Neuroimaging Initiative (ADNI) and Autism Brain Imaging Data Exchange (ABIDE) are widely used collections containing imaging data and clinical data presented in tabular format. ABIDE [165] is a dataset with over a thousand static fMRI image data. This dataset is accompanied by phenotypic data, which provide age and gender, along with scores derived from different tests, including visual IQ score, performance IQ score, handedness score, social response score, and medicine names. These data are gathered to determine whether or not an individual exhibits the characteristics of autism. Accessing and using the pre-processed version of ABIDE’s neuroimaging dataset is easier. ADNI encompasses several studies, including ADNI 1, 2, 3, and GO. These studies are focused on investigating the development of slight cognitive disability and its transition into Alzheimer’s dementia. The ADNI project manages a collection of about 7000 MRI and PET images from over 1700 people. Various non-imaging variables, including clinical and genetic data, accompany these images. ADNI also has a simpler version called TADPOLE, which is extensively utilized in medical image processing research. TADPOLE includes a selection of samples and attributes from ADNI-3. TADPOLE does not contain unprocessed images, but rather provides processed structural data related to images, like volumes of brain sub-regions, cortical viscosities, and means of regions of interest (ROIs). Additional information can be accessed on the webpage dedicated to the dataset.

The UK Biobank dataset [166] is a comprehensive collection of multimodal imaging data, including abdomen, heart, brain MRI, ultrasound, and dual X-ray scans. Additionally, it contains phenotype data and genomic information from 100,000 subjects. The data are mainly utilized for inhabitant research, particularly for examining cross-sectional relationships. Furthermore, research is conducted on the correlations between genetic variants and phenotypic imaging. Nevertheless, the creators declare that the process of collecting data is still ongoing, and the possible approaches and directions to be taken in future research have not yet been determined.

A more recent development is the introduction of RadFusion [139], a collection containing high-resolution CT scans along with electronic health records (EHRs). It identifies pulmonary embolisms by analyzing data from 1794 individuals across multiple demographic subcategories, including race, gender, and age. Finally, we would like to highlight two collections: MIMIC-III [167] and TCIA (The Cancer Imaging Archive) [168]. The MIMIC-III comprises EHRs from critical-care patients. The data elements in this dataset can be correlated with MIMIC-CXR-JPG and utilized as multimodal data. The TCIA is a compilation of medical images, including MR, CT, and nuclear medicine. Some of these images are accompanied by image analyses, medical records, and genomic data. A collection of freely accessible multimodal datasets containing imaging and structured non-imaging data is provided in Supplementary Table S4.

#### 4.2.5. Author’s Insights

In the context of multimodal fusion, it has been noted that early fusion is not commonly selected because of structural limitations. This is because early fusion requires combining unprocessed images with unprocessed structured non-imaging data. The observations indicate that the multimodal techniques regularly outperformed their unimodal equivalents. A study by Arik et al. [147] designed a framework specifically utilizing deep learning to analyze structured data. These frameworks are anticipated to eventually be incorporated into the fusion of multimodal structured and imaging data streams. However, the present level of research in this area is unclear, as indicated by references [169,170].

When it comes to understanding representations from images and structured data, contrastive learning with the assistance of patient metadata appears to be a popular and reliable option. Both references [153] present tangible proof supporting the advantages of multimodal prior training compared to unimodal prior training. The study [153] demonstrates that multimodal pre-training is more effective regarding label usage for the downstream task than fully supervised approaches. According to Table 3 it is evident that previous research only focuses on classification and retrieval tasks. It is speculated that clinical metadata might enhance the performance of other analyzing tasks, such as object detection and segmentation. When working with images and structured data, there is a noticeable absence of studies in cross-modality translation. When presented with an image, the potential for multi-task learning is a promising research area where different metadata fields can be learned simultaneously. However, the lack of studies may be due to the complex and non-linear relationship that every parameter demands, which makes learning impractical. Finally, the datasets that include each imaging and structured supplementary metadata require additional preparation to enhance their accessibility to computer researchers. According to the details in Table 4, the data compilations contain a significant amount of valuable data. However, they provide challenges in navigating and constructing the training and validation sets.

The combination of structured data and medical imaging reveals how MMML can offer a more comprehensive view of patient health. As fusion strategies evolve, their alignment with clinical needs becomes increasingly promising for real-time healthcare support.

## 5. Discussion

### 5.1. Trends in MMDL

Current trends in radiology MMDL indicate a significant shift toward more personalized and precise diagnostic methods. Integrating AI with advanced imaging techniques leads to earlier and more accurate disease detection. Multimodal machine learning is a rapidly growing area of study that is attracting more and more interest from deep learning enthusiasts [11]. In radiology, a substantial incentive exists to utilize several modalities [171,172,173] to enhance the understanding of images by incorporating additional medical information inputs. This drive provides a thorough overview of 60 papers that discuss the use of multimodal machine learning in radiography. This section offers a concise summary of our discoveries, acknowledging the constraints of this study and outlining potential avenues for further exploration in MMDL.

**Data sources**. Multimodal data sources are essential for the progression of radiology-focused machine learning, allowing models to leverage more comprehensive patient information. The MIMIC series has been transformative in this regard, offering a combination of chest X-rays, radiology reports, and electronic health records (EHRs). Through structured and unstructured data, these datasets provide a rich context that enhances model robustness, allowing insights into diverse patient care aspects, such as admission status and treatment outcomes. The success of MIMIC has underscored the importance of developing large-scale multimodal datasets across various anatomical domains. For example, cardiac, brain, or abdominal imaging could also benefit from similar multimodal datasets. By expanding beyond the thoracic focus of MIMIC, research can address a broader array of clinical needs, improving diagnostic performance and enabling disease-specific applications. However, balancing data availability with privacy is crucial for advancing multimodal machine learning in healthcare applications.

**Applications:** Multimodal machine learning applications in radiology are mainly focused on disease classification, often using fusion-based and representation-learning methods. Multimodal data have shown clear advantages in this domain by integrating data from radiographic images with patient history and clinical notes, leading to enhanced diagnostic accuracy. It aligns with clinical practices, where physicians rely on multiple sources for comprehensive decision-making. Emerging applications are exploring domain translation, such as transforming radiology images into clinical notes. This task bridges computer vision and natural language processing (NLP), utilizing models like transformers that can understand both visual and textual information. By automating this transformation, systems could assist radiologists in report generation, improve workflow efficiency, and potentially reduce clinician burnout. The evolving synergy between computer vision and NLP in radiology will likely inspire novel multimodal applications, leveraging the strengths of both fields.

**Approaches:** The taxonomy presented in this review categorizes approaches based on how multimodal data are integrated into machine learning models. The primary methods include fusion (early, joint, late), representation learning, and cross-modality translation. These techniques cover a wide range of strategies for incorporating multimodal data into supervised and unsupervised settings. CNNs are commonly used for image data, while RNN, LSTM, and transformers handle textual data. Tabular data are either directly processed through dense neural layers or used as an auxiliary input for weak supervision, such as in contrastive learning setups. By jointly learning representations, models can effectively generalize across modalities, which is crucial for clinical tasks involving structured and text data. Cross-modality translation is growing, particularly in tasks that include translating image features into text-based information. Such approaches are instrumental in report generation tasks, where a model learns to generate detailed radiology reports from imaging data alone.

It is asserted that the taxonomies outlined in this study encompass all the possible ways in which data from multiple sources can be utilized, both as the model’s input and output. It also includes both supervised and unsupervised methods, with a change in preference towards the unsupervised method. From a modal standpoint, it is evident that CNNs are the preferred choice for processing images. Regarding text, the methods used range from RNNs to LSTM networks to transformers. Tabular data are primarily analyzed utilizing dense layers after being input to a neural network. It can also be utilized for weak supervision, such as selecting negative and positive pairs for contrastive learning.

**Potential opportunities or possibilities:** Additionally, there is a notable gap in integrating temporal data (e.g., time series information like ECG or EEG) with imaging modalities. While imaging data paired with static records (e.g., EHR data) are prevalent, models that can process and learn from temporal patterns alongside imaging data would significantly enhance diagnostic accuracy and patient monitoring capabilities. This area remains relatively underexplored, possibly due to technical and clinical complexities associated with synchronizing these data types. Research that develops techniques to handle multimodal temporal data in radiology could open new avenues for patient care.

### 5.2. Limitations

In this review, the articles that best represented the challenges and advances in MMDL in radiology were carefully selected. However, there are inherent limitations associated with this selection process and scope.

**Selection Subjectivity:** Although it is aimed to provide a comprehensive overview, the selection of articles involved subjective judgments based on perceived relevance to core challenges in MMDL. This selection process inevitably introduces bias, as different researchers might prioritize alternative studies or focus on distinct aspects of multimodal research. Although we intended to provide a thorough overview, the examination of the image synthesis and image-to-structured data, and vice versa, translation corpus is not exhaustive.

**Publication Bias:** This work is also subject to publication bias—a common issue in scientific literature where positive or promising results are more frequently published than negative findings. This bias may lead to an overemphasis on the benefits of multimodal approaches, potentially skewing the perceived efficacy of MMDL methods. Consequently, the advantages of using multimodal data over unimodal data in practical clinical settings may be overestimated. A more balanced perspective, including studies with negative or inconclusive findings, would provide a more nuanced understanding of the limitations of MMDL.

**Scope of Data Modalities:** The review focused on studies incorporating image, text, and tabular data, aligning with typical clinical practices in radiology. However, this scope inherently limits the diversity of modalities considered. For example, modalities like audio, video, and time series data were excluded, as they are less commonly utilized in radiological diagnostics, but may hold potential for broader medical applications. Additionally, excluding multimodal research that does not involve imaging narrows the scope and may omit relevant insights from other medical domains where imaging is not the primary data source.

**Clinical Relevance of Multimodality:** Another limitation arises from whether multimodal approaches are universally beneficial in radiology. From an information-theoretic perspective, adding more modalities does not always guarantee improved diagnostic accuracy. For instance, radiology reports often summarize findings from an image and omit normal, non-pathological details, focusing on critical information while omitting potentially redundant data. In contrast, other data sources, such as lab results or genetic testing, may add unique insights that imaging alone cannot capture, especially for complex conditions where non-imaging data are critical for a complete diagnosis.

This review does not fully address the feasibility of improvements that could arise from integrating such complementary data sources. Adding multiple modalities could sometimes dilute the overall signal if the additional modalities provide redundant or less informative data. Conversely, multimodal approaches could be more informative and valuable in cases where modalities capture distinctly different aspects of a patient’s condition (such as combining images with genetic data or lab results). Therefore, future work should explore the potential advantages of multimodal data and the contextual limits where adding more modalities may not necessarily enhance performance. Moreover, a deeper exploration into how different modalities contribute unique information—and how to effectively combine them to minimize redundancy—could help guide future research in multimodal machine learning in radiology.

### 5.3. Future Research

Figure 1 serves as a guiding framework for understanding multimodal data’s diverse yet interconnected roles in radiology. Building on this, we highlight several high-impact research directions to overcome current limitations and advance MMML from academic exploration to clinical deployment.

**Dominance of radiographs in available datasets:** Compared to the other image formats, radiography is significantly preferred. The reason for this is the availability of hospital-scale datasets from medical professionals. However, if more medical imaging data, such as MRI and CT scans, become available in the databases, it may result in investigations that complement current studies and give a more realistic depiction of actual practices. The publication of multimodal data incorporating these new imaging modalities would expand the MMDL field, enabling researchers to investigate a larger range of clinical applications and provide conclusions more indicative of real-world diagnostic methods.

**Underutilization of non-image-centric text data:** Existing multimodal databases in radiology generally comprise image-centric text data, like radiologist reports directly related to particular imaging results. However, non-image-centric medical descriptions (e.g., medical histories, lab results, discharging reports) might contribute important additional data. These text types of information give greater context regarding a patient’s health, including details about underlying disorders, complications, and prior therapies. Incorporating non-image-centric data into multimodal evaluation might provide a more complete perspective of a patient’s state, enhancing model efficiency in challenging diagnostic situations and allowing for more individualized treatment plans.

**Inclusion of clinical time series data:** While MMDL primarily concentrates on static image, text, and tabular data, time series data (such as ECG, EEG, and continuous vital signs) in combination with imaging are absent. Time series data record dynamic physiological changes, which might be particularly valuable in forecasting patient outcomes, monitoring illness development, or giving early warning indications. The lack of open access databases containing such data may restrict research in this field. However, as more institutions deploy electronic health monitoring systems, future MMDL research might combine time series data with imaging to provide comprehensive, real-time medical surveillance.

**Expanding beyond bimodal approaches:** Although numerous current studies concentrate on bimodal combinations (usually image and text), we believe that future research will examine combinations beyond just two modalities. Instead, integrating image, tabular, and text data might give a more thorough view of a patient, leading to a greater awareness of complicated conditions and more targeted therapy suggestions. Moreover, this involves researching to obtain patient-level representations from multimodal data while addressing the lack of specific modalities. Achieving this, however, requires robust frameworks that are incapable of developing patient-level representations that compensate for absent modalities—a prevalent problem in real-world medical research.

**Challenges in evaluation and baseline comparisons:** A significant methodological difficulty in MMDL research is the establishment of suitable baselines for assessing multimodal systems. Consequently, we urge specialists to include evaluations of unimodal baselines when evaluating multimodal approaches. This will ascertain if multimodal approaches are more efficient and successful in managing sparse and noisy data prevalent in real-world situations. By continuously assessing multimodal versus unimodal baselines, researchers may obtain perspectives on the label efficiency and resilience of multimodal designs, therefore better evaluating whether their advantages surpass the associated costs in practical implementations.

In conclusion, while MMDL in radiology has significant potential, realizing its full capabilities necessitates overcoming existing restrictions and broadening research to include a wider array of data sources, more comprehensive datasets, and more stringent assessment frameworks. These developments may eventually enhance the precision, contextual awareness, and patient-centeredness of healthcare, hence improving diagnostic accuracy and patient outcomes.

## 6. Conclusions

The field of radiology is increasingly focusing on MMDL, which involves integrating multiple forms of information beyond only medical imaging to enhance medical decisions. Integrating various data modalities has led to more accurate and comprehensive diagnoses, paving the way for personalized patient care. The significance of MMDL lies in its ability to synthesize complex datasets, offering previously unattainable insights. This review has highlighted key aspects of MMDL in radiology, showcasing its potential to revolutionize medical diagnostics. This study examines MMDL and categorizes the research into two combinations: imaging with text data and imaging with structured data. Each combination is organized according to a general classification, which includes technical tasks like cross-modality retrieval, representational learning, modality fusion, and datasets. This taxonomy not only provides a structured overview of the current state of research, but also guides newcomers to the field, helping them navigate the methodological diversity and practical challenges inherent in MMML. In addition, the overview emphasizes the current areas of research that have not been addressed and the unresolved challenges that need to be overcome to facilitate upcoming development. Integrating multimodal data will likely be a game changer in medical imaging and clinical decision-making. Advances in multimodal deep learning are expected to lead to models that can generate patient-level representations encompassing a patient’s full health profile, offering a more holistic view and enabling more precise, personalized treatments. However, achieving this vision will require addressing the challenges associated with data availability, model interpretability, and the fusion of heterogeneous data types.

In conclusion, multimodal deep learning is not just a complementary approach to traditional imaging methods, but an essential step toward advancing the field of radiology and improving patient outcomes. A sound foundation is provided by this review for future research, offering insights into the current state of the art while also paving the way for the next wave of innovations in multimodal machine learning in healthcare.

## Figures and Tables

**Table 3:** Performance overview of the papers utilizing fusion strategies for imaging and structured non-imaging data comprised in this overview (MM stands for multimodal).

Reference	Framework	Dataset	Image Modality	Structured Data Modality	Fusion Strategy	Metric	MM Performance
[17]	PENet, Elastic Net	Private	CT	EHR	Late fusion	AUC	0.947
[139]	PENet, Elastic Net	RadFusion	CT	EHR	Late fusion	AUC	0.946
[140]	ResNet-50, MLP	Private	MRI	EHR	Joint fusion	AUC	0.898
[141]	3D ResNet-50, DenseNet	ADNI	MRI	EHR	Joint fusion	AUC	0.859 0.899
[142]	Multimodal 3D SiameseNet	PPMI	MRI	Clinical	Joint fusion	AUC	0.81
[144]	Inception-ResNet v2, MLP	NSCLC-Radiogenomics	CT	Clinical	Joint fusion	Accuracy AUC	0.968, 0.963
[145]	XGBoost, TabNet, SANet	Private	CT	Clinical	Joint fusion	AUC	0.96
[149]	CNN, DAFT	ADNI	MRI	Demographic Clinical	Joint fusion	Accuracy c-index	0.622 0.748
[28]	ResNet-50, XGBoost	MIMIC-CXR MIMIC-IV, JSRT	CXR	Demographic vital signs lab findings	Joint fusion	Accuracy F1 AUC	0.812 0.859 0.810
[150]	ResNet-18 + RF	NLST	CT	Clinical	Joint fusion	AUC	0.8021
[151]	CNN, NN	I-SPY-1 TRIAL	MRI	Demographic	Joint fusion	Accuracy AUC	0.83 0.80

**Table 4:** Performance overview of representation learning papers using image and structured non-image data included in this paper.

Reference	Framework	Dataset	Image Modality	Structured Data Modality	Representation Strategy	Metric	MM Performance
[153]	SimCLR + MICLe	CheXpert	CXR	Metadata	Contrastive learning	Accuracy	0.688
[154]	MedAUG	CheXpert	CXR	Metadata	Contrastive learning	AUC	0.906
[157]	Masked Siamese Network (MSN)	MIMIC-CXR CheXpert	CXR	EHR	Non-contrastive learning	AUROC	0.801
[158]	BYOL, SimCLR	Southampton Moorfields	OCT	Metadata	Contrastive learning	AUC AUC	0.85 0.88
[159]	ViT, FCNet	NSCLC	CT, PET	Metadata	Contrastive learning	C-index	0.756
[160],	PaCL	NAH dataset	CXR	Metadata	Contrastive learning	mAUC	0.836
[161]	DenseNet	BHB dataset	MRI	Metadata	Contrastive learning	AUC	0.7633

## Data Availability

No new data were created or analyzed in this study. Data sharing is not applicable to this article.

## Supplementary Materials

The following supporting information can be downloaded at: https://www.mdpi.com/article/10.3390/bioengineering12050477/s1, Table S1: Performance of literature in this review for report generation from medical imaging and text data. (MM stands for multimodal); Table S2: Performance of literature synthesizing medical images from text. IS, NIQE, SSIM, and FID stand for Inception Score, Natural Image Quality Evaluator, Structural Similarity Index, and Frechet Inception Distance respectively; Table S3: List of freely reachable multimodal datasets providing imaging and text modalities; Table S4: A collection of freely accessible multimodal datasets containing imaging and organized non-imaging data.
