# Supplementary material for: Advancements in Medical Radiology Through Multimodal Machine Learning: A Comprehensive Overview
Source: Bioengineering (Basel). 2025 Apr 30;12(5):477. doi: 10.3390/bioengineering12050477 (PMC12108733; doi:10.3390/bioengineering12050477)
Supplement: Supplementary file 1 [file bioengineering-12-00477-s001.zip › bioengineering-3565655-supplementary.pdf]

Supplementary Materials

Supplementary Table S1. Performance of literature in this review for report generation from medical imaging and text data. (MM stands for multimodal).

Reference	Framework	Dataset	Text modality	Image modality	Metrics	MM Performance
[89]	DenseNet121<br>LSTM	MIMIC-CXR	Text reports	CXR	BLEU4<br>CIDEr	0.305<br>0.850
[90]	CNN-RNN-RNN<br>Reinforcement learning	MIMIC-CXR	Text reports	CXR	BLEU1<br>CIDEr<br>ROUGE	0.352<br>1.153<br>0.307
[91]	Densenet<br>Knowledge graph	IU-X-ray	Text reports	CXR	BLEU1<br>ROUGE-L<br>METEOR	0.441<br>0.367<br>0.304
[92]	Transformer,<br>Rational memory	MIMIC-CXR<br>IU X-Ray	Text reports	CXR	BLEU1<br>ROUGE-L<br>METEOR	0.353, 0.470<br>0.277, 0.371<br>0.142, 0.187
[93]	Transformer,<br>TF-IDF	IU X-Ray	Text reports	CXR	BLEU1<br>ROUGE-L<br>METEOR	0.505<br>0.383<br>0.195
[94]	AlignTransformer	MIMIC-CXR<br>IU X-Ray	Text reports	CXR	BLEU1<br>ROUGE-L<br>METEOR	0.484, 0.378<br>0.379, 0.283<br>0.204, 0.158
[95]	Transformer,<br>Knowledge graph	MIMIC-CXR<br>IU X-Ray	Text reports	CXR	BLEU1<br>ROUGE-L<br>METEOR	0.369, 0.512<br>0.295, 0.383<br>0.153, 0.195
[97]	CNN	MIMIC-CXR	Text reports	CXR	BLEU1<br>ROUGE-L<br>METEOR	0.560<br>0.580<br>0.550
[98]	CXR-RePaiR<br>CLIP	MIMIC-CXR<br>CheXpert	Text reports	CXR	BLEU2<br>F1	0.092, 0.088<br>0.274, 0.352
[99]	ResNeXt-101<br>BERT	IU X-Ray	Text reports	CXR	BLEU1<br>BLEU4	0.580<br>0.27
[100]	Transformer,<br>Reinforcement learning	MIMIC-CXR<br>Open-i	Text reports	CXR	BLEU4<br>F1<br>Accuracy	0.114, 0.131<br>0.567, 0.483<br>0.771, 0.960
[101]	Densenet-121<br>BERT	MIMIC-CXR<br>Open-i	Text reports	CXR	BLEU4<br>ROUGE-L<br>F1	0.116, 0.139<br>0.265, 0.327<br>0.622, 0.491
[102]	LLMs<br>Transformer	MIMIC-CXR<br>IU X-Ray	Text reports	CXR	BLEU1<br>ROUGE-L<br>METEOR	0.420, 0.514<br>0.291, 0.401<br>0.167, 0.215

Supplementary Table S2. Performance of literature synthesizing medical images from text. IS, NIQE, SSIM, and FID stand for Inception Score, Natural Image Quality Evaluator, Structural Similarity Index, and Frechet Inception Distance respectively.

Reference	Framework	Dataset	Image modality	Text modality	Metrics	MM Performance
[119]	ViT-B/16 CLIP	Private	MRI	Text Reports	FID	41.35
[120]	LLM GAN	MIMIC-CXR Open-i	CXR	Text Reports	FID NIQE	8.821, 5.745 4.113, 4.113
[121]	RoentGen Stable Diffusion 2.1	RSNA	CXR	Text Reports	AUC Dice mAP	0.899 (class) 0.698 (segm) 0.249 (detec)
[122]	cGAN LSTM	MIMIC-CXR Open-i	CXR	Text Reports	IS FID SSIM	1.112, 1.081 86.15, 141.5 0.379, 0.343
[117]	ViT, BERT	ImageCLEF 2019, SLAKE, VQA-RAD, ROCO	Radiology scans	Clinical questions	ACC	0.799 0.833 0.835 0.785

Supplementary Table S3. List of freely reachable multimodal datasets providing imaging and text modalities.

Dataset name	Text modality	Image modality	URL
MIMIC-CXR	Radiological Reports	CXR	*"https://physionet.org/content/mimic-cxr/2.0.0"*
MIMIC-CXR-JPG	Radiological Reports	CXR	*"https://physionet.org/content/mimic-cxr-jpg/2.0.0"*
IU X-ray	Radiological Reports	CXR	"*https://openi.nlm.nih.gov/* *https://paperswithcode.com/dataset/iu-x-ray"*
PADCHEST	Radiological Reports	CXR	*"https://bimcv.cipf.es/bimcv-projects/padchest"*
RadGraph	Radiological Reports	CXR	*"https://physionet.org/content/radgraph/1.0.0"*
COV-CTR	Radiological reports	CT	*"https://github.com/mlii0117/COV-CTR"*
COVID-19 CT	Radiological reports	CT	*"https://covid19ct.github.io"*
CANDID-PTX	Radiological Reports	CXR	*"https://doi.org/10.17608/k6.auckland.14173982"*
CheXpert	Radiological Reports	CXR	*"https://stanfordmlgroup.github.io/competitions/chexpert"*
NIH-CXR	Radiological Reports	CXR	*"https://nihcc.app.box.com/v/ChestXray-NIHCC"*
RSNA	Image captions	CXR	*"https://rsna.org/challenge-datasets/2018"*
ImageCLEF	Image Captions	Diverse	*"https://www.imageclef.org"*
MEDICAT	Image Captions	Diverse	*'https://github.com/allenai/medicat"*
ROCO	Image Captions	Diverse	*https://github.com/razorx89/roco-dataset"*
SLAKE	Clinical questions	Radiological images	*"https://www.med-vqa.com/slake"*
VQA-RAD	Clinical questions	Radiological images	*"https://osf.io/89kps"*

Supplementary Table S4. A collection of freely accessible multimodal datasets containing imaging and organized non-imaging data.

Dataset name	Publication	Structured Non-image modality	Image Modality	No, of Studies
UK Biobank	[169]	Cross-sectional	MRI, X-ray, Ultrasound	100,000
RadFusion	[142]	Cross-sectional	CT	1794
ABIDE I	[172]	Cross-sectional	MRI	1,112
ABIDE II	[168]	Cross-sectional	MRI	2,156
ADNI	-	Longitudinal	MRI	> 7,000
MIMIC-III	[170]	Longitudinal	CXR	-
TCIA	[171]	Both	MRI, CT, Nuclear	-
